# Directed Network Motifs in Alzheimer’s Disease and Mild Cognitive Impairment

**DOI:** 10.1371/journal.pone.0124453

**Published:** 2015-04-16

**Authors:** Eric J. Friedman, Karl Young, Graham Tremper, Jason Liang, Adam S. Landsberg, Norbert Schuff, for the Alzheimer's Disease Neuroimaging Initiative

**Affiliations:** 1 International Computer Science Institute, Berkeley, CA, United States of America; 2 Department of Computer Science, University of California, Berkeley, Berkeley, CA, United States of America; 3 Department of Radiology & Biomedical Imaging, University of California San Francisco, San Francisco, CA, United States of America; 4 VA Medical Center, San Francisco, CA, United States of America; 5 W.M. Keck Science Department, Claremont McKenna College, Pitzer College, and Scripps College, Claremont, CA, United States of America; & National Laboratory of Pattern Recognition, CHINA

## Abstract

Directed network motifs are the building blocks of complex networks, such as human brain networks, and capture deep connectivity information that is not contained in standard network measures. In this paper we present the first application of directed network motifs in vivo to human brain networks, utilizing recently developed directed progression networks which are built upon rates of cortical thickness changes between brain regions. This is in contrast to previous studies which have relied on simulations and in vitro analysis of non-human brains. We show that frequencies of specific directed network motifs can be used to distinguish between patients with Alzheimer’s disease (AD) and normal control (NC) subjects. Especially interesting from a clinical standpoint, these motif frequencies can also distinguish between subjects with mild cognitive impairment who remained stable over three years (MCI) and those who converted to AD (CONV). Furthermore, we find that the *entropy* of the distribution of directed network motifs increased from MCI to CONV to AD, implying that the distribution of pathology is more structured in MCI but becomes less so as it progresses to CONV and further to AD. Thus, directed network motifs frequencies and distributional properties provide new insights into the progression of Alzheimer’s disease as well as new imaging markers for distinguishing between normal controls, stable mild cognitive impairment, MCI converters and Alzheimer’s disease.

## Introduction

The study of networks constructed from human brain imaging has been providing new insights into the architecture of the brain as well as new tools to understand the changes that arise in a wide variety of brain disorders [1,2]. These networks are often referred to as connectomes and their study as connectomics [2]. Their nodes consist of brain regions and the edges represent pairwise relationships between these regions, such as physical connections from dMRI tractography, functional connections from fMRI correlations, or even anatomical correlations among cortical thickness measurements over time, as used in this paper [2–4].

Abnormal brain network patterns have been shown to have potential diagnostic value [1,2]. However, brain networks constructed from in vivo imaging data are extremely noisy. While global network properties, such as average path length or average clustering coefficient, are fairly similar among normal subjects and relatively stable for individuals over time, individual edges can be quite unreliable [5,6], preventing the use of a single or even a small number of edges, such as those in a localized brain region, in most analyses. Thus, we focus on global (averaged) measures as is common in such analyses that often construct structural networks based on tractography from dMRI or functional networks from fMRI [2].

In this paper we consider an approach that captures “local structures” in a brain network known as directed network motifs, but averages them over the entire network to improve statistical robustness. Thus, even without trusting any specific edge or motif, one can capture local structures, “on average.” Directed network motifs were originally developed for the analysis of protein networks and related applications [7,8]. For brain networks, directed network motifs have previously been used in vitro to understand the brain structure of macaques and cats [9,10]. Simulations of directed network motifs have also been used to study human brain structure [2]. To our knowledge, directed network motifs have not been previously applied to in vivo human brain networks. In addition they appear to be important for understanding the dynamical behavior of neuronal networks, such as the synchronization of neuronal clusters [11,12].

In order to construct these directed network motifs we apply a recently developed protocol called “directed progression networks” (DPNets) which utilizes longitudinal brain MRI data to construct edges which capture the disease progression over time, separately for each subject. DPNets were introduced in Friedman et al. [3], which studied their basic properties and showed their utility for distinguishing cognitive normal subjects from patients diagnosed with Alzheimer’s disease (AD) at both the group and individual level. Directed network motifs are the building blocks of complex networks, such as arise in the brain, capturing deep connectivity information that is not contained in standard network measures.

**Fig 1:**
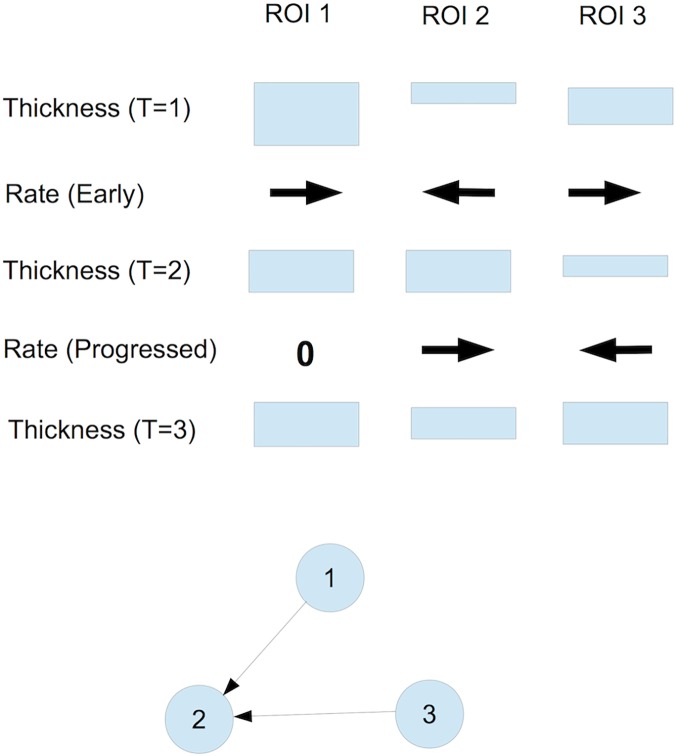
DPNet construction. Schematic DPNet Construction for three regions-of-interest (ROIs) based on three yearly (T = 1,2,3) MRIs. ROI 1 thins in the early period (large positive thinning rate) and remains unchanged (0 rate) in the progressed period. ROI 2 thickens in the early period and thins in the progressed period. ROI 3 thins in the early period and thickens in the progressed period. Thus, the DPNet has edges from 1→2 and 3→2 showing the thinning progression and possible disease progression. However, even though the rate in the early period for ROI 2 is similar to the rate for ROI 3 in the progressed period, there is no edge from 2 to 3, since a negative thinning rate is not evidence of a diseased ROI. Intuitively, we see that ROIs 1 and 3 are thinning in the early period, which implies that they are diseased early, while ROI 2 appears diseased in the progressed period and may have been infected by ROI 1 or 3 or perhaps both, which is represented by the directed edges.

DPNets are constructed from the rates of cortical thickness changes in brain regions, attempting to find signals of disease progression which is indicated by thinning rates. See Fig 1 for an overview of their construction and Friedman et al. [3] for more details. This study used data from the Alzheimer’s Disease Neuroimaging Initiative (ADNI). The Freesurfer automated labeling system for subdividing the human cerebral cortex on MRI scans into gyral based regions of interest as well as labeling subcortical regions was used for extracting 88 regions-of-interests (ROI).

In this paper we demonstrate the utility of DPNets in brain MRI, both as a source of insights into the development of AD as well as potential imaging markers of the disease. We show how DPNets can capture aspects of “large-scale system disruptions” in the AD anatomical network, as discussed in Xie and He [13], as opposed to local disruptions from single region analyses. In particular, the directed network motifs can be used to characterize the statistical complexity of the spread of the disease across brain regions. We show that several key motifs can differentiate between normal brain networks and those arising from brains with AD. In addition, we find that the *entropy* of the motif distribution [14]—a measure of the variety of motifs present—provides additional useful information and, most notably, appears to be a new and interesting imaging marker of cognitive decline toward AD, i.e. in non-demented subjects who show signs of mild cognitive impairment (MCI).

## Methods

### Subjects

This study used data from 255 subjects (39 AD, 65 MCI, 54 CONV, and 97), from the Alzheimer’s Disease Neuroimaging Initiative that were publicly available at the time of the data analysis. All subjects had at least three 1.5T MRI scans taken at least every year which were successfully evaluated using Freesurfer software version 4.4 [15,16]. At the end of the 3 years study period, each subject had a diagnosis consistent with Alzheimer’s Disease (AD), stable mild cognitive impairment (MCI), MCI conversion to AD (CONV), or stable normal cognition (NC). MCI is a clinical concept that characterizes cognitive stage intermediate between normal aging and AD [17].

The ADNI was launched in 2003 by the National Institute on Aging (NIA), the National Institute of Biomedical Imaging and Bioengineering (NIBIB), the Food and Drug Administration (FDA), private pharmaceutical companies and non-profit organizations, as a $60 million, 5-year public-private partnership. The primary goal of ADNI has been to test whether serial magnetic resonance imaging (MRI), positron emission tomography (PET), other biological markers, and clinical and neuropsychological assessment can be combined to measure the progression of mild cognitive impairment (MCI) and early Alzheimer's disease (AD). Determination of sensitive and specific markers of very early AD progression is intended to aid researchers and clinicians to develop new treatments and monitor their effectiveness, as well as lessen the time and cost of clinical trials. The Principal Investigator of this initiative is Michael W. Weiner, MD, VA Medical Center and University of California San Francisco. ADNI is the result of efforts of many co-investigators from a broad range of academic institutions and private corporations, and subjects have been recruited from over 50 sites across the U.S. and Canada. The initial goal of ADNI was to recruit 800 adults, ages 55 to 90, to participate in the research, approximately 200 cognitive normal older individuals to be followed for 3 years, 400 people with MCI to be followed for 3 years and 200 people with early AD to be followed for 2 years. For up-to-date information, see www.adni-info.org.

Subjects whose diagnosis reverted over three years, e.g. reversion from MCI to NC or AD to MCI, were excluded. See Table 1 for a summary of the demographic and clinical data of the subjects. (The full subject list, by code names, can be found in the supplementary materials, and can be used to retrieve the MRI and clinical data from the ANDI website hosted by LONI, http://adni.loni.ucla.edu/).

**Table 1:** Group demographics and clinical summary.

	NC	MCI	CONV	AD	p-value^(a)^
N	97	65	54	39	
% Male	48	56	56	55	0.12
APOE ε4 carriers (%)	33	40	48	51	< 0.001
Age (years)	75.4±5	75.0±5	74.7±5	74.5±6	0.21
ADAS-Cog11^(b)^	5.6±2	9.5±4	12.9±5	18.1±6	< 0.001
**% Annual Change** ^**(c)**^ ADAS-Cog11	-8±80	7±53	26±47	42±34	< 0.001

(a) P-values indicate effects across groups using analysis of variance (ANOVA) or Fisher exact test (for categorical variables, e.g. male and APOE) (b) Alzheimer’s Disease Assessment Scale-cognitive subscale with 11 items (Mohs et al. 1997); total score range from 0–70; larger scores indicating greater impairment. (c) % Annual change is expressed relative to baseline in percent.

The details of ADNI standard image acquisition and processing by Freesurfer are described in detail in [16,18], but we note that the acquisition consisted of T_1_-weighted MRI scans with an acquisition matrix size of 256 x 256 x 166 in the *x*-, *y*- and *z*-dimensions with a nominal voxel size of 0.94 x 0.94 x 1.2 mm using a sagittal volumetric magnetization prepared rapid gradient echo (MP-RAGE) sequence, with an echo time (TE) of 4 ms, repetition time (TR) of 9 ms, flip angle of 8°. Image quality was assessed by a designated center that corrected the data for system-specific image artifacts [18]. To estimate cortical thickness, Freesurfer computed the shortest distance from each point on the gray/white matter surface to the pial surface and averaged the result with the distance in the reverse direction. The confounding effect of intra-subject morphological variability is reduced in Freesurfer version 4.4 by using a longitudinal workflow that estimates brain morphometry measurements unbiased toward any timepoint by building first a template image from all time points as an unbiased prior distribution for each subject before computing morphometric deformations for all time points. This strategy reduces the random variation in the processing procedure and improves the robustness and sensitivity of the overall longitudinal analysis. Such an initialization scheme makes sense also because a longitudinal design is often targeted at detecting small or subtle changes within subjects.

### Directed Progression Network Reconstruction

The construction of the directed progression network followed the protocol described in [3] which constructs a personalized network for each subject. It uses a panel of longitudinal thickness measurements, extending a construction developed by Li et al. [19] for undirected networks that in turn built on research which used a single temporal measurement to construct a single (undirected) network based on a group of subjects [4,20,21]. The key idea in this protocol is to capture the spread of the “disease” in the brain through the pattern of thinning cortical regions over time.

As in the previous study, our construction is based on three timepoints taken at one-year intervals, from which we compute “thinning rates” in the *early period* (i.e., between timepoints 1 and 2) and in the *progressed period* (i.e., between timepoints 2 and 3). If a specified node is thinning in the *early* period (and hence potentially “diseased”) and this thinning rate is determined to be correlated with the thinning rate in the *progressed* period of a *second* node, then in our network construction we create a directed edge from the first node to the second node. The resulting network captures the notion that the disease may have potentially “spread” from the first node to the second. (See Fig 1 for a schematic description.)

**Fig 2:**
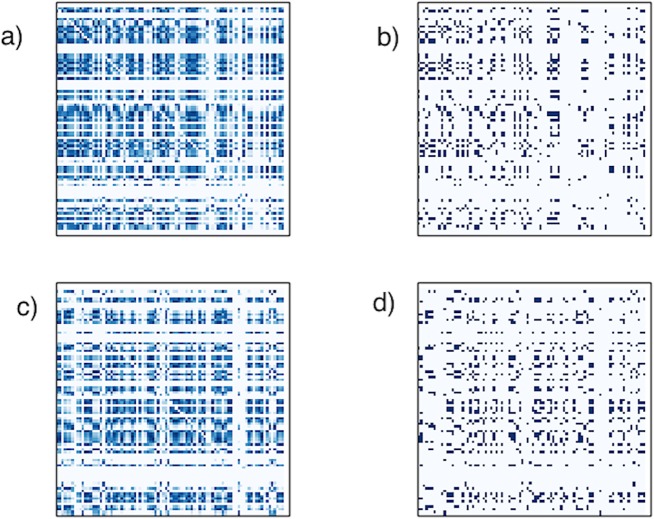
DPNet matrices. (a) Connectivity heat map for sample NC patient DPNet matrix. Note that it is not symmetric, as the connections are directed and has horizontal bands that reflect nodes that might be infected in the early period. (b) Matrix representation of thresholded DPNet network for NC patient. (c) Connectivity heat map for sample AD patient DPNet matrix. (d) Matrix representation of thresholded DPNet network for NC patient.

Since each node in our network construction is a standard anatomical ROI, the directed networks we work with all have 88 nodes, which include subcortical regions. The number of edges can vary depending upon the choice of a threshold (as is typical in brain network constructions [2]); however in this paper we choose individual thresholds for each subject such that the directed networks of all subjects have exactly 880 edges (yielding an average outdegree of 10). (As is standard in most network analyses [2], we do not allow for self edges (edges from a node to itself).) This procedure whereby we individually select the threshold for each subject so as to maintain a constant average degree is significant, since motif distributions are highly dependent on the number of edges, and thus this procedure controls for the exogenous source of noise in the comparison of different subjects’ networks. The exact choice of the average outdegree, 10, was chosen to be consistent with other studies and provide maximum information as discussed in more detail in [3]. Analyses with other average outdegrees yield substantially similar results and are discussed in the results section. Fig 2 shows heatmaps for one NC and one AD patient both before and after thresholding.

### Motif Analysis

**Fig 3:**
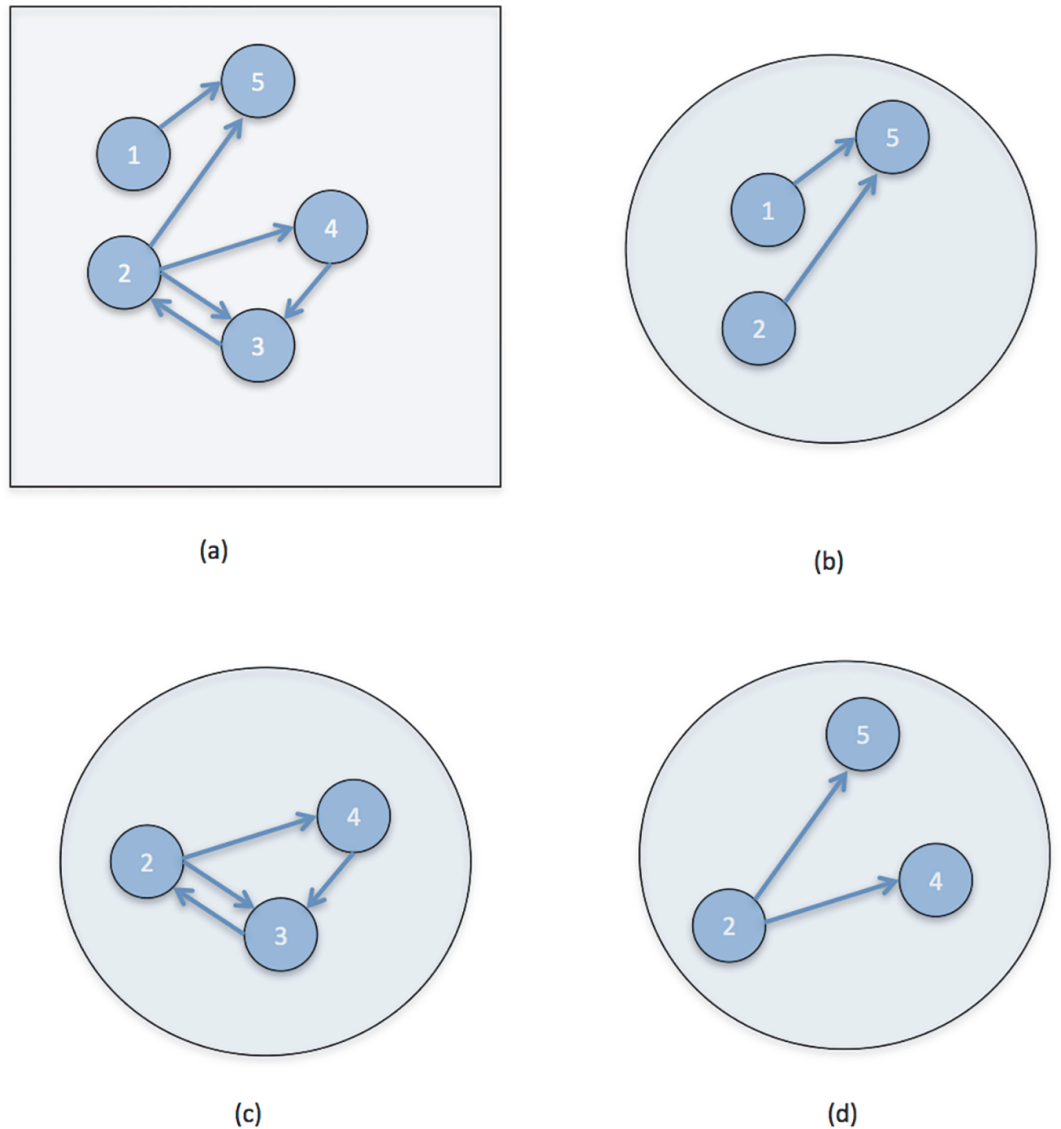
Sample construction of directed network motifs. A sample network is shown in part (a), while three of its 3-node subnetworks are shown in (b)-(d). (The motif with nodes 2,3,5 is not shown.) Note that triplets of nodes that are not part of a connected subnetwork are not considered motifs, such as the node triplet (1,3,5).

**Fig 4:**
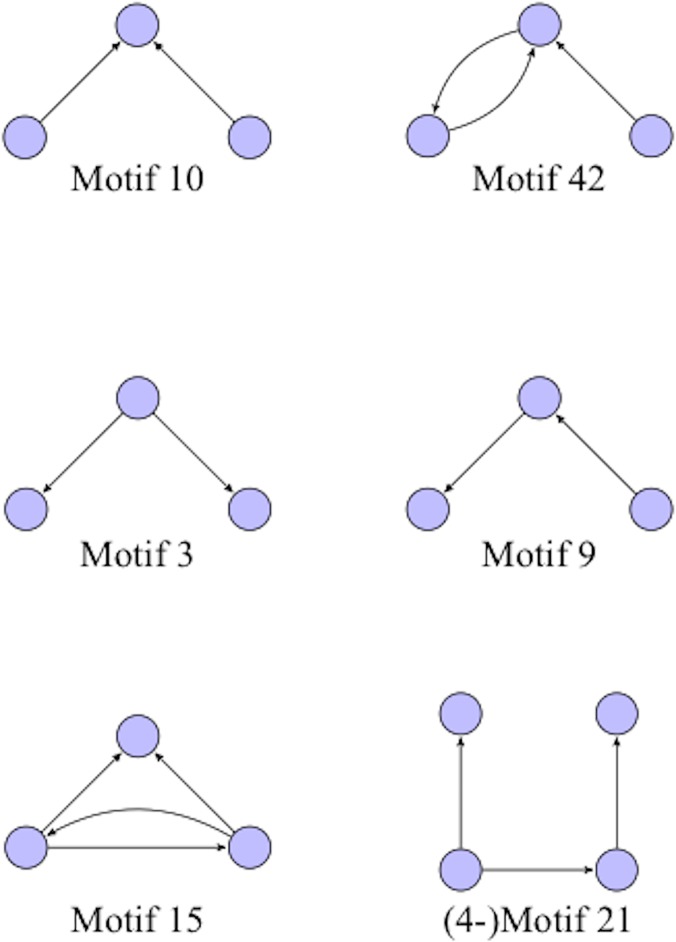
Representative motifs. Representative 3-node motifs (and one 4-node directed motifs). The motif ID numbers are those used by the Kavosh software (Kashani et al. 2009). Note that the motif ID numbers for different values of k can be duplicates, i.e. the same motif number can refer to both a 2-motif and a 3-motif.

Our analysis focuses on directed motifs, a sensitive indicator of the local structure in directed networks [8,9,22]. A k-motif is a small, connected, k-node sub-network of the original network, as shown in Fig 3. For example, in a directed network there can be at most 13 distinct directed 3-motifs and 199 distinct directed 4-motifs, several of which are shown in Fig 4. To find the motifs in a network we used the Kavosh software package [23], which exhaustively computes the numbers of each type of k-motif in a network (here we employ the same motif labeling scheme used in that paper). At the top level, this algorithm is quite simple. It finds all subnetworks of size k and then puts them into groups with the same network structure. The details are much more complex, as Kavosh uses sophisticated tree-based data structures to find all subnetworks efficiently and then applies a state of the art algorithm (NAUTY) based on canonical labeling to compare subnetworks.

Note that only connected triplets of nodes are considered in the analysis of 3-motifs, which significantly reduces the total number of motifs in a sparse network. For example, three nodes that only have a single edge between a single pair of them do not form a 3-motif. Nonetheless, the number of possible k-motifs still grows exponentially with k, limiting the analysis to small values of k. Since k-motifs for small k, as employed in this study, only use information from a small number of nodes at a time, the numbers and types of potential motifs do not depend significantly on the size or details of the network. See Fig 4 for some examples of specific motifs, including those that will play a large role in our analysis.

As noted, to label motifs we will use the motif ID labeling scheme defined in the Kavosh software package and described in [23]. Each ID uniquely identifies a specific k-motif and is based on a lexicographic ordering of the adjacency matrix for the motif. (See Fig 4.)

We considered the fraction of different k-motifs (the number of each specific motif type normalized by the total number of k-motifs found) for each the different types of subjects, AD, CONV, MCI, and NC for k = 3–6. In addition we also assessed the distribution of k-motifs using the entropy (ENT), a measure of distribution uniformity [14]. If there are m different motifs 1,2,…m and the fraction of motif j is p_j_ then the entropy of the distribution is given by:

$$\text{ENT}=-({\text{p}}_{1}\;\text{log}({\text{p}}_{1})+{\text{p}}_{2}\;\text{log}({\text{p}}_{2})+\ldots +{\text{p}}_{\text{m}}\;\text{log}({\text{p}}_{\text{m}}))$$

which is maximized when all motifs are equally likely (in which case ENT = log(m)), and minimized when there is only a single motif (ENT = 0); thus decreased entropy indicates a reduction in the variety of motifs and/or a more asymmetric distribution of existing motifs.

We used a non-parametric permutation testing procedure to assess statistical significance. For each motif frequency or motif entropy, the individual subjects DPNets association with the diagnosis groups (NC, MCI, CONV, or AD) was randomly mixed 5000 times to estimate the distribution of t-values. We then calculated the two-tailed P-values from this distribution.

For each network we also computed the corresponding expected motif distributions for several matched random networks. In particular, we considered both fully random Erdos-Renyi (ER) networks [24] with the same number of edges and degree-distributed random networks (DD) [25] which match the precise degree distribution of the original network,. To compute the DD networks we used 1000 “double edge swaps” and “directed triangle orientation reversals.” The former are well known from undirected networks, while the latter “directed triangle orientation reversals” are required to successfully randomize directed degree distributed random networks [26].

## Results

### 3-Motif Frequencies

**Fig 5:**
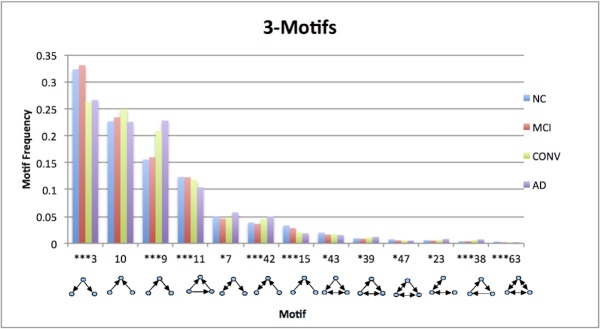
Motif frequencies for NC, MCI, CONV and AD. Significance refers to NC/AD comparisons.

The average 3-motif frequencies for the most significant motifs are shown in Fig 5. While the rank ordering of the motifs is fairly consistent among all subject types, significant differences in their motif frequencies are observable between normal control (NC) subjects and AD patients for most of the common motifs. In fact, twelve of the top thirteen 3-motifs are statistically significant for separating NC from AD (each at the 5% confidence level), with the majority of these significant at the 0.1% level. Hence these constitute new imaging markers for distinguishing AD from NC.

As can be seen in Table 2 the motif frequencies do not distinguish well between AD and MCI, as there are only three 3-motifs which are statistically significant at the 5% level when uncorrected for multiple comparisons and not significant when corrected. This also arises in the CONV vs. AD comparison, although the similarity between these two groups is expected since CONV subjects probably have some pre-clinical AD condition already at baseline. In contrast, MCI vs. CONV comparison has four 3-motifs which are statistically significant at the 1% or 0.1% levels. Thus we see a progression from NC to MCI to CONV to AD with statistical significance as we cross from MCI to CONV.

**Table 2:** Disease progression P-values for 3-motifs.

Motif	NL/AD	NL/MCI	MCI/CONV	CONV/AD
3	0.007	0.366	0.001	0.404
10	0.468	0.301	0.156	0.046
9	0.000	0.363	0.001	0.074
11	0.000	0.463	0.171	0.013
7	0.015	0.142	0.256	0.003
42	0.006	0.241	0.006	0.169
15	0.000	0.089	0.013	0.259
43	0.017	0.029	0.332	0.179
39	0.016	0.202	0.024	0.136
47	0.020	0.036	0.392	0.448
23	0.047	0.214	0.258	0.174
38	0.000	0.386	0.004	0.092
63	0.000	0.012	0.186	0.214

Two-tailed P-values (non-Bonferroni corrected) for 3-motifs ordered by NC motif frequency, for the most common motifs. (The full list is not shown due to space constraints.)

Intriguingly, the frequencies of motifs numbers 9 and 42, which imply respectively sequential spread and feedback interaction (see Fig 4), tend to progressively increase from NC to AD, whereas the frequency of motif number 3, which implies a seed-based parallel spread, declines, as discussed in the next section.

### 4-Motif and 5-Motif Frequencies

Given the large number (~200) of 4-motifs, we will consider Bonferroni [27] corrected p-values in this section, in order to be very conservative in our findings.

The 4-motifs (Table 3) provide new imaging markers as there are twenty-eight 4-motifs which are significant (Bonferroni corrected) at the 5% level for distinguishing NC from AD, NC from CONV, and, more interestingly from a clinical standpoint, MCI from CONV and MCI from AD. The situation is more subtle for distinguishing NC from MCI, which is not surprising given that not all MCI will develop AD but may simply represent an extreme of normal aging [28]. In this case there are no motifs that are significant at the 5% (Bonferroni corrected) level, but there are 17 that are significant at the 5% uncorrected level. This suggests that these are not random occurrences as the probability of this occurring at random is less than 3% using the fact that the number of false positives is Poisson distributed for independent events [29]. This arises as the Bonferroni corrections are designed to prevent a single uncorrected factor from being spuriously significant, but when a large number appear significant then it is likely that several are truly significant.

**Table 3:** 4-Motif frequencies.

Motif ID	NC Frequency	NC/AD	NC/MCI	MCI/CONV	AD/CONV
21	0.072	< 0.001	0.230	0.016	0.027
164	0.062	< 0.001	0.417	< 0.001	0.340
14	0.033	< 0.001	0.371	0.025	0.012
161	0.028	< 0.001	0.419	< 0.001	0.273
162	0.017	< 0.001	0.177	< 0.001	0.175
267	0.005	< 0.001	0.440	0.010	0.043
2196	0.004	< 0.001	0.328	0.010	0.167
142	0.003	< 0.001	0.169	0.007	0.043
2190	0.001	< 0.001	0.299	0.002	0.309
678	0.000	< 0.001	0.256	0.053	0.034
2188	0.000	< 0.001	0.290	< 0.001	0.352
54	0.048	< 0.001	0.068	< 0.001	0.023
55	0.040	< 0.001	0.308	0.001	0.035
63	0.016	< 0.001	0.235	0.004	0.197
270	0.015	< 0.001	0.425	0.001	0.360

4-Motif Frequencies and (non-Bonferroni corrected) p-values for most common motifs for the CORR and ICORR DPNets. (The full list is not shown due to space constraints.)

For the 5-motifs there are approximately 4000 different motifs with at most frequency 0.03, which makes statistical significance difficult to achieve a seen in Tables 4, 5 and 6.

**Table 4:** 5-Motif frequencies by NC.

Motif	NC Frequency	NC/AD	NC/MCI	MCI/CONV	AD/CONV
15	0.047	0.282	0.483	0.286	0.107
1098	0.030	0.010	0.047	0.189	0.403
45	0.026	0.011	0.110	0.314	0.053
206	0.023	0.029	0.316	0.458	0.094
142	0.019	0.014	0.322	0.437	0.037
105	0.018	0.042	0.121	0.463	0.358
33416	0.018	0.072	0.353	0.201	0.326
2188	0.016	0.024	0.233	0.235	0.424
239	0.015	0.090	0.391	0.500	0.132
238	0.015	0.026	0.428	0.322	0.097

5-Motif Frequencies and (non-Bonferroni corrected) p-values for most common motifs sorted by NC motif frequency. (The full list is not shown due to space constraints.)

**Table 5:** 5-Motif frequencies by NC/AD.

Motif	NC Frequency	NC/AD	NC/MCI	MCI/CONV	AD/CONV
268889	5.45E-05	< 0.001	0.0598	0.1034	0.101
136108	1.59E-06	< 0.001	0.0644	0.0022	0.1448
72364	3.34E-06	< 0.001	0.116	0.4598	0.0136
135884	2.36E-04	< 0.001	0.1208	0.1674	0.0762
541123	1.63E-05	< 0.001	0.1456	0.2542	0.039
139726	9.05E-06	< 0.001	0.1644	0.1756	0.015
82988	1.02E-04	< 0.001	0.1776	0.462	0.0062
34357	1.76E-04	< 0.001	0.2012	0.048	0.1936
135852	1.42E-05	< 0.001	0.2828	0.1448	0.015
295734	8.87E-07	< 0.001	0.2838	0.2072	0.0908

5-Motif Frequencies and (non-Bonferroni corrected) p-values for most common motifs sorted by NC/AD p-values. (The full list is not shown due to space constraints.)

**Table 6:** 5-Motif frequencies by NC/MCI.

Motif	NC Frequency	NC/AD	NC/MCI	MCI/CONV	AD/CONV
38606	1.67E-06	0.0072	0.0002	0.0052	0.0318
545599	1.89E-07	0.0962	0.0002	0.0818	0.2732
36014	2.64E-04	0.2686	0.0002	0.0016	0.4594
104142	2.03E-07	0.0098	0.0006	0.001	0.0162
166061	7.82E-07	0.1636	0.0006	0.004	0.3524
148373	4.72E-07	0.3078	0.0008	0.1016	0.1892
50107	3.45E-05	0.0758	0.0012	0.3074	0.1038
169437	3.25E-06	0.2392	0.0012	0.0708	0.4174
100670	2.89E-07	0.266	0.0012	0.0014	0.2938
543918	2.55E-05	0.4208	0.0012	0.0934	0.1416

5-Motif Frequencies and (non-Bonferroni corrected) p-values for most common motifs sorted by NC/MCI p-values. (The full list is not shown due to space constraints.)

There are no significant (Bonferroni corrected) motifs at the 5% level; however, there are approximately 600 5-motifs that are significant for distinguishing NC from AD at the (uncorrected) 5% level and 241 at the (uncorrected) 0.02% level. These 600 motifs have a negligibly small probability of arising by chance, using the fact that the number of false positives is Poisson distributed for independent events [29], which implies that most of the 600 uncorrected significant 5-motifs are truly imaging markers and not random chance.

### Motif Distributions

The entropy of the k-motif distribution is a global statistic over the set of all k-motifs found in a specific DPNet and is shown in Table 7.

**Table 7:** Motif entropies by Type.

	NC	MCI	CONV	AD
3-Motifs	1.759	1.723	1.804	1.833
4-Motifs	3.471	3.408	3.572	3.627
5-Motifs	5.427	5.344	5.628	5.724

The motif entropy is smallest and statistically significant at the 1% level for distinguishing MCI from CONV and AD for 3-motifs, 4-motifs and 5-motifs, as seen in Table 8. (All p-values for entropies are non-Bonferroni corrected.) The other comparisons are not statistically significant, although the separation of NC to AD is statistically significant at the 5% level for 5-motif entropy and at the 10% level for 3-motif and 4-motif entropies. However, one sees a clear progression in the data where the entropy falls from NC to MCI then rises from MCI to CONV and from CONV to AD.

**Table 8:** Motif entropy P-values.

Significance vs MCI (P-values)			
	NC	CONV	AD
3-Motifs	0.353	0.018	0.006
4-Motifs	0.427	0.018	0.008
5-Motifs	0.518	0.012	0.004

Significance vs. MCI.

### Comparisons with Reference Networks

To elucidate the basis for our main results we compared the motif frequencies and entropies between the subject DPNets and standard matched random networks, specifically the Erdos-Renyi (ER) and Degree Distributed (DD) networks. As seen in Table 9 there is a significant difference (typically less than 0.02% significance) between 3-motifs of the subject DPNets and the matched Erdos-Renyi networks, showing that the underlying DPNets are clearly not random in the ER sense. (Note that p-values in this section are not Bonferroni corrected.) For the degree distributed (DD) matched random networks, the comparison is more subtle. For some of the 3-motifs the DD and subject DPNets are not significantly different, while many key motifs differ for the NC patients. However, only one 3-motif differs for the DD networks and the matched AD DPNets.

**Table 9:** Reference network P-values for 3-motifs.

Motif	NC/ER(NC)	NC/DD(NC)	AD/DD(AD)	AD/ER(AD)
3	< 0.001	0.395	0.426	< 0.001
10	0.004	0.330	0.368	< 0.001
9	< 0.001	0.274	0.437	< 0.001
11	< 0.001	0.498	0.256	< 0.001
7	0.075	0.340	0.427	0.012
42	< 0.001	0.216	0.458	0.217
15	< 0.001	0.004	0.101	< 0.001
43	< 0.001	< 0.001	0.046	< 0.001
39	< 0.001	0.038	0.046	< 0.001
47	< 0.001	0.401	0.224	< 0.001
23	< 0.001	0.016	0.025	< 0.001
38	< 0.001	0.347	0.322	< 0.001
63	< 0.001	< 0.001	0.013	< 0.001

Two-tailed P-values (non-Bonferroni corrected) for 3-motifs ordered by NC motif frequency, for the most common motifs for DPNets vs. reference networks. Note that, for example, ER(NC) refers to Erdos Renyi Networks which are matched to the average edge probability of the NC subjects while ER(AD) are matched to the AD subjects.

For the 4-motifs (table not shown due to space considerations) the pattern is more apparent. Here one can identify strong differences between both the ER and DD matched networks for the NC patients, although the results are weaker for the AD patients and their matched networks. Nonetheless in these cases the matched random networks differ significantly from the subjects’ DPNets. The matched ER networks differ significantly from the subjects’ DPNets while the DD networks differ on a significant fraction of the motifs for both the NC and AD patients.

For the 5-motifs (table not shown due to space considerations), though the patterns may appear suggestively similar to those for the 4-motifs, the small size of the individual motif frequencies precludes a meaningful quantitative analysis.

### Varying the average degree

The motif frequencies for average degree 8, 10 and 12 are shown in Table 10. Note that the ordering of the most common motifs is mostly unchanged by changes in average degree. Also, as seen in Table 11, the statistical significance for the motifs in distinguishing between NC and AD are mostly unchanged for different average degrees.

**Table 10:** Motif frequencies.

Motif	NC(8)	NC(10)	NC(12)	AD(8)	AD(10)	AD(12)
3	0.3251	0.3228	0.3222	0.2757	0.2661	0.2584
10	0.2447	0.2266	0.2083	0.2396	0.2255	0.2119
9	0.1787	0.1555	0.1373	0.2530	0.2280	0.2068
11	0.1097	0.1232	0.1327	0.0889	0.1042	0.1179
7	0.0443	0.0496	0.0523	0.0513	0.0577	0.0620
42	0.0363	0.0387	0.0401	0.0444	0.0500	0.0530
15	0.0234	0.0333	0.0449	0.0120	0.0188	0.0261
43	0.0149	0.0199	0.0242	0.0099	0.0155	0.0205
39	0.0077	0.0095	0.0109	0.0092	0.0122	0.0154
47	0.0047	0.0076	0.0108	0.0024	0.0052	0.0085
23	0.0044	0.0059	0.0071	0.0058	0.0081	0.0099
38	0.0039	0.0039	0.0039	0.0072	0.0074	0.0072
63	0.0020	0.0034	0.0053	0.0006	0.0013	0.0024

Motif frequencies for NC and AD and average degrees of 8, 10 and 12. Note that the motifs are ordered by their NC(10) frequencies.

**Table 11:** Significance for NC vs. AD.

Motif	NC(8) vs. AD(8)	NC(10) vs. AD(10)	NC(12) vs. AD(12)	NC(8) vs. NC(10)	NC(8) vs. NC(12)	NC(10) vs. NC(12)
11	***	***	**	**	***	**
10	-	-	-	-	**	-
39	-	*	***	*	***	-
38	***	***	***	-	-	-
15	***	***	***	**	***	**
23	-	*	*	**	***	-
47	**	*	-	***	***	**
42	*	**	**	-	-	-
43	**	*	-	**	***	**
3	*	**	**	-	-	-
63	***	***	***	**	***	**
7	*	*	**	*	**	-
9	***	***	***	*	***	*

Table of p-value significance for comparisons between NC and AD for average degrees of 8,10 and 12, and NC with itself for different average degrees. Legend: >5% (-), 1–5% (*), 0.1–1% (**), <0.1% (***).

However, as one can see in Table 11, the motif frequencies are strongly dependent on the average degree. For example, even comparisons between the NC group with degree 8 and itself with degree 12 are statistically significant at the 0.1% level for 10 of the top 13 motifs.

## Discussion

Our primary finding is that directed network motifs provide potentially powerful new imaging markers for distinguishing NC or stable MCI subjects from AD patients or MCI subjects who developed AD (CONV) over the 3 years study period. We believe these results are encouraging for a prognosis of AD and could be further strengthened with the use of additional longitudinal data.

### Motif Frequencies

First we note that 3-,4- and even 5-motifs provide new imaging markers for distinguishing NC subjects from AD patients or CONV patients and—more notably from a clinical perspective—MCI from CONV. Some motifs even achieve statistical significance for the more challenging distinction between NC and MCI subjects or between CONV and AD patients. (Although a direct comparison is difficult, using DPNet motifs appears to provide somewhat stronger classification than using thinning rates of specific regions, such as the Hippocampus [30].)

The statistics for the 4-motifs and 5-motifs is rather subtle but informative. As discussed earlier, although most motifs are not significant when corrected for multiple tests using the stringent Bonferroni criteria, the number of motifs that are significant without correction (at p < 0.05) is very statistically significant. This is because the Bonferroni correction is chosen to prevent a single false positive significant uncorrected motif but is not correct for multiple uncorrected positives. Extending the Bonferroni calculation to multiple uncorrected positives shows that many of those are very likely to be statistically significant [29]. The difficulty is that one cannot tell which of those are the significant ones, thus qualitative analysis of the specific motifs (as in the seed-based discussion above) is quite subtle and have not been taken on in this work. However, for imaging markers one can use the set of potentially significant motifs to create a highly statistically significant imaging marker in a straightforward manner by controlling for the false discovery rate (FDR) as in [29].

The comparison with matched random ER networks shows that these results rely on underlying structural aspects of the networks and not just average thinning rates. Furthermore, the comparisons with matched random DD networks suggest that while much of this structure depends on the degree distribution of the underlying DPNets, it is not the whole story. It appears that motifs are providing a tractable mechanism for capturing complex correlations between edges of the networks, which is otherwise difficult to quantify.

Beyond the direct application of motifs as statistical imaging markers, the details of the motifs themselves may provide insights into the spread of Alzheimer’s disease across the brain. Intriguingly, but speculatively, the structure of the 3-motifs numbers 9 and 42 (see Fig 4) are suggestive of, respectively, sequential spread (the “infection” progresses along a path of nodes) and feedback interaction (progression cycles between two nodes). Observe that the frequencies of these two motifs both progressively increase from NC to AD, whereas the frequency of 3-motif number 3, which is suggestive of a seed-based parallel spread (i.e., a single node appears to be spreading the “infection” among two of its network neighbors), declines. One can interpret this in terms of disease stages, implicating changes in the mode of disease spread as the severity of clinical symptoms progresses.

The analysis of the motif entropies complements this detailed motif analysis, showing a progression: MCI is the most structured (i.e., lowest entropy, most non-uniform), and AD the least structured (i.e. highest entropy, most uniform). This not only provides a new and interesting imaging marker for MCI but also suggests that the critical spreading stage of the disease is the most structured. This provides statistical support for the intuition that in AD most regions are affected so there is more uniformity in edges. This is a potentially important finding in the broader effort to understand the mechanisms of Alzheimer’s disease propagation, which is thought to systematically spread across the brain. The increase in motif entropies is also consistent with the proposed progression of the AD pathology, which is believed to start in nuclei of the brainstem, then progresses toward the entorhinal cortex and hippocampus before spreading more diversely to neocortical regions [31]. Furthermore, increasing entropy, manifested through a complex network of age-related molecular changes, is also considered an etiologic principle toward the transition from normal age-related cognitive decline to sporadic AD [32]. Motif entropies could therefore also be useful for disease staging.

Another interesting aspect of our analysis is the effectiveness of 4- and 5-motifs as statistically significant imaging markers, despite the large underlying statistical fluctuations associated with them. Thus, their statistical significance appears to be possible both due to the size of the ADNI data set as well as perhaps underlying properties of the directed progression networks, which allow the motifs to capture signals from the patterns of disease spread. In fact, our analysis of these networks was based on Bonferroni corrected statistics [33], which are quite conservative compared to the more commonly used false discovery rate. We therefore believe that the results for these higher degree motifs are robust and potentially even stronger than stated above. It will be interesting to learn through future studies whether higher-order motifs provide additional insight in high noise levels arising from the large numbers of such motifs.

### Varying the average degree

As seen in Tables 10 and 11, while our results are stable for changes in average degree, comparing networks with different average degrees. Thus, we believe that one should use fixed degrees in the analysis of motifs, when there is no clear biological reason for setting a specific fixed threshold. One could then capture variable average degree effects by other measures, such as the sum of all cells in unthresholded DPNets.

### Directions for Future Work

Our finding that the frequency of network motifs can provide significant new markers for Alzheimer’s disease at the group level suggests a number of new or related avenues or research. The extension to markers at the individual level, which is more challenging but important from a diagnostic point of view, is currently being investigated. In addition, the observation that motifs of primarily sequential progressions seem to increase in frequency from NC to AD, whereas the frequency of those of parallel progressions decline, intriguingly suggests that directed network motifs could help to better understand the mechanisms how AD pathology spreads across the brain. Studies of regional variations in directed motif patterns in conjunction with imaging studies mapping AB-amyloid, a hallmark of AD, might provide additional clues about the spread of the disease or might be useful for the assessment of effective treatment interventions. In particular, the change in network structure for MCI and its value to predict which MCI subject remain stable and which will ultimately develop AD is an important topic of further study.

In addition there are many other directions to extend our analysis. For example, correlating motifs with patient characteristics, such as age, gender and genetic markers is an important direction of study. Another important direction would be to examine the spatial location of the motifs. This is complicated both by the nonlocality of the motifs but more importantly by the loss of statistical significance by localizing the analysis which significantly reduces the size of the “sample” as only a small fraction of the motifs are located in any specific region.

Lastly, we note that our methods can be directly applied to other neurodegenerative disease that are thought to progressively spread across the brain, since DPNets could similarly capture the progression patterns of the neuro-degeneration, which vary by disease. For instance, progressive supranuclear palsy, corticobasal degeneration, and frontotemporal dementia each has distinct patterns of degeneration [34–38]. Furthermore in [39] it is shown that pattern of thinning for Alzheimer disease and frontotemporal dementia can be modeled as the action of a network diffusion operator on the brain’s structural network. This suggests that one might be able to use the patterns of the DPNets for these diseases to understand their temporal dynamics of these disease progressions.

A few limitations of the present study ought to be considered as well: Since AD was not confirmed by autopsy, the exact contribution of AD pathology to variations in motif structures remains unclear. A limited clinical ability to identify progressors in the NC and MCI groups over the three-years follow-up, i.e. those who convert after 3 years, may have skewed the motif distributions of these two groups. A technical limitation is that by analyzing anatomical regions to keep computations tractable we implicitly made the assumption of a homogenous propagation of thinning with each region. A finer analysis of cortical thinning, e.g. voxel-by-voxel, may modify the results.

## Supporting Information

S1 Text List of Subjects by ADNI subject code. (TXT)
